# *Pycard* and *BC017158* Candidate Genes of *Irm1* Locus Modulate Inflammasome Activation for IL-1β Production

**DOI:** 10.3389/fimmu.2022.899569

**Published:** 2022-06-21

**Authors:** Andrea Borrego, Francesca Colombo, Jean Gabriel de Souza, José Ricardo Jensen, Alice Dassano, Rocco Piazza, Barbara Anaís Rodrigues dos Santos, Orlando Garcia Ribeiro, Marcelo De Franco, Wafa Hanna Koury Cabrera, Marcelo Yudi Icimoto, Nancy Starobinas, Geraldo Magalhães, Leticia Figueiredo Monteleone, Silas Fernandes Eto, Carlos DeOcesano-Pereira, Mauricio Barbugiani Goldfeder, Kerly Fernanda Mesquita Pasqualoto, Tommaso A. Dragani, Olga Célia Martinez Ibañez

**Affiliations:** ^1^ Laboratory of Immunogenetics, Instituto Butantan, São Paulo, Brazil; ^2^ Department of Research, Fondazione Istituto di Ricovero e Cura a Carattere Scientifico (IRCCS), Istituto Nazionale dei Tumori, Milan, Italy; ^3^ Centre of New Target Discovery (CENTD), Instituto Butantan/GlaxoSmithKline (GSK)/Sao Paulo Research Foundation (FAPESP), São Paulo, Brazil; ^4^ Department of Medicine and Surgery, University of Milano-Bicocca, Milan, Italy; ^5^ Diagnosis Center, Instituto Pasteur, São Paulo, Brazil; ^6^ Laboratory of Biophisics, Federal University of São Paulo, São Paulo, Brazil; ^7^ Laboratory of Immunopathology, Instituto Butantan, São Paulo, Brazil; ^8^ Laboratory of Development and Innovation, Instituto Butantan, São Paulo, Brazil; ^9^ Development and Innovation Laboratory, Center of Innovation and Development, Instituto Butantan, São Paulo, Brazil; ^10^ ALCHEMY – Inovation, Research & Development Ltd., University of São Paulo, São Paulo, Brazil

**Keywords:** Inflammation, genome screening, cancer, ASC specks, Interleukin-1, Interleukin-6, *Pycard*, BC017158

## Abstract

We identified *Pycard* and *BC017158* genes as putative effectors of the Quantitative Trait locus (QTL) that we mapped at distal chromosome 7 named *Irm1* for Inflammatory response modulator 1, controlling acute inflammatory response (AIR) and the production of IL-1β, dependent on the activation of the NLRP3 inflammasome. We obtained the mapping through genome-wide linkage analysis of Single Nucleotide Polymorphisms (SNPs) in a cross between High (AIRmax) and Low (AIRmin) responder mouse lines that we produced by several generations of bidirectional selection for Acute Inflammatory Response. A highly significant linkage signal (LOD score peak of 72) for *ex vivo* IL-1β production limited a 4 Mbp interval to chromosome 7. Sequencing of the locus region revealed 14 SNPs between “High” and “Low” responders that narrowed the locus to a 420 Kb interval. Variants were detected in non-coding regions of *Itgam*, *Rgs10* and *BC017158* genes and at the first exon of *Pycard* gene, resulting in an E19K substitution in the protein ASC (apoptosis associated speck-like protein containing a CARD) an adaptor molecule in the inflammasome complex. Silencing of *BC017158* inhibited IL1-β production by stimulated macrophages and the E19K ASC mutation carried by AIRmin mice impaired the *ex vivo* IL-1β response and the formation of ASC specks in stimulated cells. IL-1β and ASC specks play major roles in inflammatory reactions and in inflammation-related diseases. Our results delineate a novel genetic factor and a molecular mechanism affecting the acute inflammatory response.

## Introduction

Inflammation is a complex process involved in the evolution of several diseases, such as Alzheimer, cancer, asthma, metabolic, cardiovascular and chronic rheumatic diseases and in aging (1–5). Despite several molecular mechanisms of inflammation being well known, such as processes related to the recruitment of immune and inflammatory cells, the release of chemical mediators (such as cytokines) and tissue repair, the genetic mechanisms involved in inflammation development and progression are still not clear (6–8).

To investigate this point we make use of AIRmax and AIRmin mouse lines that we obtained after several generations of bidirectional phenotypic selective breeding, on the basis of acute inflammatory reactivity (AIR) (9). The selection phenotypes for the inflammatory response were the number of infiltrated leukocytes and exudated proteins content in local inflammatory exudate, harvested 24h after subcutaneous injection of polyacrylamide beads (Biogel). At the end of the selection, the phenotypic divergence between the two lines reached about 30 fold in the number of infiltrated cells and 2.5-fold in proteins concentration in inflammatory exudates, as a result of the differential accumulation of alleles with opposite effects at various Quantitative Trait Loci (QTLs) influencing the acute inflammation intensity in each line.

Previously in order to map these loci, we carried out a SNP based whole genome linkage study in a population of 290 (AIRmax x AIRmin)F2 mice obtained by crossing AIRmax and AIRmin mouse lines. We identified one major quantitative trait locus (QTL) on chromosome 7 named *Inflammatory response modulator 1* (*Irm1*) which spanned about 15 Mbp and contained 230 genes (10). This locus modulates inflammatory phenotypes such as leukocyte influx in inflammatory exudates and in particular the interleukin-1beta (IL-1β) production by blood leukocytes after NLPR3 inflammasome activation (10).

In the present study, with the aim of identifying *Irm1* candidate genes, we tested *Irm1* locus association with IL-1β levels and other inflammation phenotypes in a larger (AIRmax x AIRmin)F2 pedigree comprising about 700 mice. To investigate the molecular bases of inflammation we looked for single nucleotide polymorphisms (SNPs) in 4 genes within the *Irm1* locus that were possibly involved in the modulation of IL-1β production and/or of acute inflammation. Results of the present study will help in the identification of genes and molecular mechanisms that modulate the intensity of the inflammatory response.

## Materials and Methods

### Mice and Treatments

AIRmax and AIRmin lines (formally designated Ibut : AIRH and Ibut : AIRL at the Institute for Laboratory Animal Research, National Research Council) and crosses were developed and maintained at the animal facilities of the Laboratory of Immunogenetics of the Butantan Institute, São Paulo, Brazil. An (AIRmax × AIRmin)F2 mice population, consisting of 693 mice (352 males and 341 females) was generated as described (9). AIRmin sublines were produced by genotype assisted mating for the fixation of *Pycard* C and T alleles in homozygosity.

#### Quantification of the Inflammatory Response

For *in vivo* acute inflammatory response, pouches were formed in a previously shaved dorsal region of mice by sc injection of 750 µL sterile suspension of 67% Biogel P100 (Biorad) (53mg dry weight/mL) in phosphate-buffered saline (PBS). Local infiltrated leukocytes were harvested after 24h and quantified by total cell counting in Malassez chambers. Cell free supernatants were used for IL-6 quantification by ELISA.

For *ex vivo* IL-1β production by inflammasome activation, whole heparinized blood (250 μL) was diluted in 200 μL RPMI 1640 medium supplemented with 1% FCS (Vitrocell), gentamycin (Sigma Aldrich) and inflammatory cells present in blood samples were stimulated to produce IL-1β with 50 μL *Escherichia coli* LPS (1 μg/mL) (Sigma-Aldrich) for 3h at 37°C. Then, 5 mM ATP (Sigma-Aldrich) was added and blood samples were incubated for another 1h at 37°C, to allow for IL-1β release from cells. Blood samples were centrifuged at 400 × g for 10 min to remove cells and collect supernatants containing IL-1β. Secreted IL-1β levels in plasma and IL-6 levels in inflammatory exudates were measured with the OptEIA Mouse IL-1β or Mouse IL-6 ELISA kits (BD Biosciences, San Diego, CA), respectively.

#### Caspase 1 Activation

Mice were sacrificed by CO_2_ inhalation and then the peritoneal cavity was washed with 5 mL of PBS. The cell suspension was washed twice with PBS by centrifugation and the concentration was adjusted to 10^6^ cells/mL. Cells 2 x 10^6^ per well were distributed in 6-well plates for adhesion (about 1 to 2 h) at 37°C 5% CO_2_. The supernatant was discarded, and the cells were removed with a cell scraper. Cell extracts were prepared by suspending 10^6^ cells in 500 μL of 50 mM Hepes buffer (pH 7.4) and lysed by sonication (40s, 40Hz). Cell debris were pelleted for 5 min at 12000 g, and the supernatants were used for protein quantification (Bradford) and Caspase-1 assay. Briefly, 100 μL of supernatants were added to 1mL of 50 mM Hepes buffer (pH 7.4) plus 5 mM DTT, in a 1 cm cuvette at 37°C. The substrate (10 μM Abz-YVADNQ-EDDnp), was added, and the fluorescence was measured using a spectrofluorimeter (F2500, Hitachi co., Japan) under agitation for 30 min and the slope was normalized as total protein in each point as relative fluorescence units (RFU/min.μg). The specific inhibitor Ac-YVAD-CMK (5μM) was also tested to ensure substrate specificity.

#### Carcinogen Treatment

Groups of male and female mice (30 to 40 in each group) were injected ip with 300 mg/Kg bw urethane (Carbamic acid ethyl ester, Sigma-Aldrich, St Louis MO) seven days after birth. Mice were euthanized 270 days later for analysis of incidence, multiplicity and volume of lung and liver tumors.

All procedures were approved by the Institutional Animal Care and Use Committee of Butantan Institute (protocol n° 3587190320), and all animals received humane care, according to the criteria outlined in the “Guide for the Care and Use of Laboratory Animals” prepared by the National Academy of Sciences and published by the National Institutes of Health.

#### Histopathology

Lung and liver tissue fragments were collected and fixed in 10% formalin buffer for 24 h. After fixation, the samples were dehydrated in alcohol (70%, 80%, 90% and 100%), diaphanized in xylene, embedded in paraffin and the blocks sectioned with a thickness of 5 µm. The slides were mounted and stained with hematoxylin - eosin (H&E) and by special staining with Periodic Acid Schiff (PAS).

### Genome-Wide SNP Genotyping and Phenotype Evaluation

Herein we take advantage of previous genotyping data of (AIRmax × AIRmin)F2 mice [described in reference (11)]. The phenotypes under investigation were the number of leukocytes and the concentration of IL-6 in inflammatory exudates harvested 24h after Biogel P100 sc injection, and IL-1β production, measured in mouse blood samples as previously described (9, 10).

#### Deep Sequencing of the *Irm1* Locus

We selected 16 (AIRmax X AIRmin)F2 mice showing extreme high or low IL-1β levels after LPS + ATP treatment. Genomic DNA was extracted from tail tips of these 16 mice with E.Z.N.A. Tissue DNA kit (Omega Bio-Tek Instruments, Watford, UK) and quantified by fluorimetry with the Quant-iT PicoGreen dsDNA quantification kit (Invitrogen, Carlsbad, CA, USA). Deep custom Illumina sequencing of the *Irm1* locus (from 124 to 128 Mb) in these 16 genomic DNA samples was carried out. The samples were analyzed using the following bioinformatics pipeline: 1) Conversion of the sequencing data from bcl to qseq; 2) Conversion of the qseq files to Sanger fastq; 3) Alignment of the fastq to the Mouse genome reference (GRCm38/mm10); 4) Filtering of the alignment data (Sam) for reads mapped in proper pair; 5) Conversion to a Binary alignment file (Bam); 6) Generation of Pileup data; 7) Pileup filtering using the following criteria: read coverage >= 10; variant coverage >= 3; variant ratio >= 0.2; mapping quality >= 30 (individual variants); read quality >= 30 (individual variants); 8) Merging of filtered Pileup data with SNPs from dnSNP137. Almost 100% of the 4Mb region was covered and the average read depth was around 75-80.

### Cell Culture and Treatment for IL-1β Release Measurement

The mouse macrophage cell line J774A.1 (ATCC^®^ TIB­67™), kindly provided by Dr. S. Recalcati (Università degli Studi di Milano, Milan, Italy) was cultured in DMEM (Lonza) supplemented with 2 mM L-glutamine (Lonza) and 10% Fetal Bovine Serum (PBI). Cells were treated for 3h with 1 μg/mL LPS (Sigma-Aldrich) to stimulate IL-1β production and for 1h with 2.5 mM ATP (Sigma-Aldrich) to induce IL-1β secretion into the supernatant. IL-1β levels in supernatants were measured by ELISA using Quantikine^®^ Mouse IL1-β kit (R&D Systems Europe Ltd., Abingdon, UK). The optical densities were detected in the Tecan ULTRA microplate reader (absorbance wavelength: 450 nm; wavelength correction for optical imperfections of the plate: 570 nm; Tecan Group, Mannedorf/Zurich, Switzerland).

### Quantitative Real-Time PCR (RT-qPCR)

Total RNA was extracted from the J774.1 cell line with the RNeasy Midi Kit (Qiagen, Valencia, CA, USA). Total RNA was purified with the RNeasy MinElute Cleanup, (Qiagen) and quantified in the Nanodrop ND-1000 Spectrophotometer (NanoDrop products, Wilmington, DE, USA). Integrity of the total RNA was evaluated using the RNA 6000 Nano Assay Kit (Agilent Technologies, Palo Alto, CA).

J774.1 RNA (1 µg) was reverse-transcribed to cDNA with the Transcriptor First Strand cDNA Synthesis Kit (Roche, Basel, Switzerland) according to the manufacturer’s instructions. Intron-spanning primers were designed for cDNA sequences of BC017158 (*BC017158*), integrin subunit alpha M (*Itgam*), regulator of G-protein signaling 10 (*Rgs10*), and hypoxanthine phosphoribosyl transferase 1 (*Hprt1*), as reference gene. Twenty-five ng of J774.1 cDNA was used in qPCR using Fast SYBR^®^Green PCR Master Mix (Applied Biosystems) with 300 nM gene-specific PCR primers (**Supplemental Table 1**) or TaqMan^®^ universal master mix no amperase UNG (Applied Biosystems). Relative expression levels were calculated using the comparative Ct method using one of the synthesized cDNA samples as calibrator.

### TaqMan Genotyping of *Irm1* SNPs

Custom TaqMan SNP Genotyping Assays (Applied Biosystems, Foster City, CA) were designed for four of the SNPs identified in a 420 kb region (**Table 1**) of the *Irm1* locus (**Supplemental Table 2**). The assays for each SNP were run in a StepOne Plus real-time thermocycler (Applied Biosystems) according to the manufacturer’s instructions, using the TaqMan™ Genotyping Master Mix and 10 ng genomic DNA per sample. DNA from mice carrying homozygous or heterozygous *Pycard* genotypes were used as controls.

**Table 1:** Deep sequencing of the *Irm1* locus in 16 (AIRmax x AIRmin)F2 mice with extreme phenotypes for the IL-1β production.

Position	Phenotype group Numbers per Genotype		*P*-value^#^	FDR	SNP	Gene	Gene region
	HighIL1β	LowIL1β					
127993599	8CC	5CT;3TT	7.49E-08	7.36E-05	NA*	***Pycard***	coding
128019362	8CC	5CT;3TT	7.49E-08	7.36E-05	NA*	NoGene	/
128025368	8AA	4AG;4GG	7.49E-08	7.36E-05	rs254560907	NoGene	/
128029428	8CC	4CA;4AA	7.49E-08	7.36E-05	rs21219299	NoGene	/
128089822	8CC	4CT;4TT	7.49E-08	7.36E-05	rs50943650	***Itgam***	intron
128106432	8GG	4CC;4GC	7.49E-08	7.36E-05	rs26084205	***Itgam***	intron
128117516	8AA	4AT;4TT	7.49E-08	7.36E-05	rs32042651	***Itgam***	utr-3
128226371	8GG	4GA;4AA	7.49E-08	7.36E-05	rs47642836	NoGene	/
128276629	8TT	4TA;4AA	7.49E-08	7.36E-05	rs23194730	**BC017158**	intron
128310294	8GG	5AA;3GA	7.49E-08	7.36E-05	NA*	NoGene	/
128312770	8TT	8 DC	4.60E-09	6.34E-05	rs24047017	NoGene	/
128356485	8CC	7C/DT;1DT	7.49E-08	7.36E-05	NA*	NoGene	/
128359768	8TT	7T/DA;1DA	7.49E-08	7.36E-05	rs23254339	NoGene	/
128414876	8GG	4GA;4AA	7.49E-08	7.36E-05	rs32381191	***Rgs10***	intron

*No annotated SNP available at this position.

^#^Association P-values of each SNP with the IL-1β levels from the tested F2 mice.

D, one base deletion; FDR, false discovery rate. Genes are highlighted in bold.

### Gene Silencing

Expression of *BC017158*, *Itgam, Pycard* and *Rgs10* genes was silenced in J774A.1 (ATCC^®^ TIB­67™) cells using specific siRNAs. Gene-silencing was carried out following a reverse-transfection protocol using the HiPerFect Transfection Reagent (Qiagen, Cambridge, MA, USA). In detail, a combination of four siRNA for each gene (Qiagen, **Supplemental Table 1**) was spotted in 48-well plates. For the *Pycard* gene an additional unique siRNA (**Supplemental Table 1**, Thermo Fisher Scientific) was used, since the pool of the four Qiagen siRNAs did not silence the expression of this gene. HiPerFect reagent (2 µL), diluted in serum free medium, was added to the pre-spotted siRNAs and incubated for 10 min at room-temperature to allow formation of the transfection complex. Sixty-thousand cells per well were seeded on top of the siRNA-transfection reagent complexes into a final volume of 120 µL. Cells were incubated at 37°C in an atmosphere of 5% CO_2_ for 6h and then fresh medium was added to each well for a final siRNA concentration of 25 nM (6.25 nM each oligonucleotide). AllStars Negative Control siRNA (Qiagen) was used as a scrambled siRNA control. At 72h after transfection cells were treated for IL-1β production, as described above; supernatants were collected and used in the ELISA whereas total RNA was extracted from cells and reverse-transcribed using the FastLane Cell cDNA kit (Qiagen) in order to verify, by RT-qPCR, the actual silencing of target genes. RT-qPCR analysis was performed using the QuantiFast^®^ SYBR^®^ Green and QuantiTect^®^ Primer Assays (Qiagen, **Supplemental Table 1**) following the manufacturer’s instructions. *Hmbs* was used as a reference to normalize expression levels. Reactions were run in duplicate on the real-time PCR 7900HT system (Applied Biosystems). Gene silencing and ELISA were repeated three times, **Supplemental Figures 1, 2A**.

### Production of J774A.1 Cells Stably Expressing WT and Mutated ASC Forms Linked to GFP

J774A.1 cells (ATCC^®^ TIB­67™) were purchased from BCRJ (Rio de Janeiro Cell Bank).

Single-guide RNA design for CRISPR/Cas9 gene editing: We used the sgRNA design software from Addgene to knock out the whole *Pycard* gene. The templates consisted of 200 nt DNA sequences of the 3800 bp ENSMUST00000033056.4 transcript each spanning 100 nt of either 5’ or 3’ UTR sequence plus 100 nt of upstream or downstream sequence, respectively. The best guides considering the PAM sequence in the gene, necessary for Cas9 activity and with the lowest off-target scores are presented in **Supplemental Figure 2A**. The diluted oligos were annealed and phosphorilated with 1μl (10U) T4 PNK kinase (New England Biolabs) in T4 ligation buffer (LB) containing ATP (Adenosine 5’-triphosphate, 10mM - New England Biolabs), in a PTC-200 thermocycler (MJ Research, Waltham, USA) with the following parameters: 37°C for 30 min, 95°C for 5 min and ramp down to 25°C at 5°C min^-1^. The phosphorylated and annealed oligos were diluted 1:200 for cloning into LentiCRISPR v2 plasmid (Addgene) (50 ng/mL), using 2 μL oligo duplex, 10mM DTT (Thermo Fisher Scientific), 10mM ATP, 10U BsmB1 enzyme (Esp3I (BsmBI) Thermo Fisher Scientific), 1500 U T7 ligase (New England Biolabs), 2 μL of 10X Tango buffer and H_2_0 for a final volume of 20 μL. We included a no-insert control replacing oligos with water (12). The reaction was treated with PlasmidSafe ATP-dependent exonuclease (Epicentre, Illumina) (37°C for 30 min, followed by 70°C for 30 min) to digest the residual linearized DNA. Competent *E coli* Stbl strain (One Shot Stbl3 chemically competent *E. coli* -Thermo Fisher Scientific) was used for plasmid transformation on ice for 10 min, 42°C for 30 sec and ice for 2 min, suspended in SOC medium and plated in LB plate containing 100 μg/mL ampicillin and incubated overnight at 37°C. Insert containing colonies were individually cultured in LB medium with ampicillin and after overnight incubation at 37°C, plasmid DNA was purified with Charge Switch Pro Plasmid Miniprep Kit (Invitrogen). Insertion was confirmed by Sanger sequencing in an ABI 3500 Genetic Analyzer (Applied Biosystems) from U6 promoter using the forward primer: hU6F – 5’ GAGGGCCTATTTCCCATGATT (Position 1 to 22) and the Reverse primer: CMV enhancer – 5’ GGGCCATTTACCGTAAGTTATG (Position 445 to 467).

Lentivirus production: an insert containing LentiCRISPR v2 (1.64 pmol) or control pLJM1-EGFP, pCMV-VSV-G (0.72pmol) and psPAX2 (1.3 pmol) (purchased form Addgene) were transfected into HEK293T cells (10^6^ cells per well in 6 well plates) using Lipofectamine +P3000 (Invitrogen). After 5h 30min medium was changed to DMEM (high glucose, pyruvate – Thermo Fisher Scientific) supplemented with Glutamax (GlutaMaxTM Supplement - Thermo Fisher Scientific) and 10% Fetal Bovine Serum (Vitrocell). Viral suspensions were collected after 48 and 72h incubation and filtered in 0.45 μM PVDF membranes.

Transduction of J774A.1 cells: In an ice bath, 1 mL of each viral preparation and 2 μL polibrene (Hexadimethrine bromide – Sigma Aldrich) (4 mg/mL) were added to 1 mL of cells in suspension (5x10^5^/mL). After spinning down for 5 min/1000 rpm, the mixture was plated in 6 well plates, and incubated for 24h at 37°C/5% CO_2_. Media was changed to complete DMEM medium and 3 days later a previously determined dose of 2 μg/mL puromycin (Puromycin Dihydrochloride - Thermo Fisher Scientific), was added for selection of transduced cells, since the transfer plasmid contains the puromycin resistance gene. After Single cell sorting in FACSAria equipment (BD Biosciences), the clones were expanded in DMEM plus 20%FCS, pyruvate, non-essential amino acids, and 2ME. PCR analysis using primers external to the guides detected 8 clones showing deletion of the *Pycard* gene^-^. (**Supplemental Figure 2B**). In 3 of the clones, we confirmed heterozygous deletion by sequencing and TaqMan genotyping assay (**Supplemental Figures 2C–E**).

#### Generation of WT ASC-GFP and E19K ASC-GFP Retrovirus and Transduction of J774 ASC^+/-^ Cells

Genes were synthetized at Genone Biotechnologies (Brazil) and cloned into pLJM1-GFP plasmid (Addgene 19319) in a way that GFP was linked to the C-terminal portion of *Pycard* (Sequences in **Supplemental Figure 3**). Viral preparations containing each of the two *Pycard* alleles were produced in HEK293T cells and transduction of J774 ASC^+/-^ with the two alleles was performed as described above. GFP-positive cells were sorted in FACSAria and suspensions were cultured in complete DMEM 10% FCS. (**Supplemental Figure 4**).

### Analysis of ASC Specks Formation in AIRmax and AIRmin PBMC

Whole blood from AIRmax and AIRmin mice was obtained by cardiac puncture. Three animals per group were used to obtain a 3 mL pool of blood for PBMC isolation with Ficoll-Paque (density 1.007 g/mL). Briefly, 3 mL of blood was carefully added to 1.5 mL of Ficoll-Paque (Ficoll^®^ Paque Plus Sigma-Aldrich) in a conical tube (5mL). After centrifugation (2300 rpm, 20 min, room temperature, w/o breaking) the PBMC layer was washed twice with PBS and split into two equal parts for inflammasome activation as described above. After LPS + ATP stimulation the reaction was stopped by fixing the cells with 4% PHEM-PFA buffer (paraformaldehyde, PHEM buffer: 60 mM PIPES; 25 mM HEPES; 10 mM EGTA; and 2 mM MgCl2, pH 6.9) (Thermo-Fisher) followed by 4% PHEM-PFA 0,1% Triton 100X for permeabilization. The cells were washed with PHEM-glycine for removal of PFA and blocked for 30 min at room temperature with 1% PHEM-BSA. The primary ASC antibody (MERCK cod. 04-147) was incubated overnight (1:250) at 4°C in an orbital shaker. After 2 washes with PBS, the secondary antibody AF647 (1:500) (Invitrogen cod. A21236) was added together with 5μM Hoechst (Thermo cod. 33342) and incubated for 2 hours at room temperature. Ten thousand events of the stained samples were acquired in an Amnis ImageStreamX MKII (Luminex). Images were analyzed using image-based algorithms in the ImageStream Data Exploration and Analysis Software (IDEAS 6.2 - Luminex) (13, 14).

### High Content Screening Analysis of ASC Specks Formation in J774A.1 Cells Overexpressing WT or Mutated ASC-GFP Constructs

Cells 2x10^4^/well were seeded in 96 well plates in a 100 μL volume and incubated at 37°C 5% CO_2_. After 24h plates were placed in a confocal microscope (ImageXpress Micro Confocal High-Content Screening (HCS) Imaging System (Molecular Devices) for analysis of real-time formation (time lapse) of ASC specks, after sensitization of cells with LPS and stimulation with ATP. Photos were recorded every 5 minutes up to 2 hours after the addition of ATP.

### Molecular Modeling - Computational Site Directed Mutagenesis Analysis

The three-dimensional (3D) structure and assembly of the mouse ASC inflammasome by combined NMR spectroscopy and cryo-electron microscopy was retrieved from Protein Data Bank, PDB (URL rcsb.org); (15)), entry code 2N1F (16), and the monomer 2N1H:A coordinates were used as template to mutate the amino acid residue Glu (polar, negatively charged), at the nineteenth position, to Lys (polar, positively charged) (E19K), in the N-terminal region (pyrin domain, PYD). The Met1 residue at the N-terminus portion was added. The molecular geometry was optimized, and partial atomic charges were assigned using the CHARMM force field (17). The energy minimized models, wild and mutated monomers, were used as input data for generating the molecular surfaces. The soft molecular surfaces were calculated using a faster and more approximate algorithm than that used for creating a solvent surface. For large molecules, this kind of surface provides better performance. The surfaces were colored according to the interpolated charges (intense blue: positively charged; intense red: negatively charged; white: non-charged) (**Figure 6A**) (Discovery Studio Visualizer 4.0; Accelrys Software Inc., 2005-2013).

### Statistics

Data were analyzed using ANOVA with Tukeys posttest for values with a normal distribution, or *Mann-Whitney U* test for comparison of two populations as indicated in the figure legends. *P*-values <0.05 were considered significant.

## Results

### Reduction of Inflammatory Response Modulator 1 (Irm1) Locus Candidate Region

**Figure 1:**
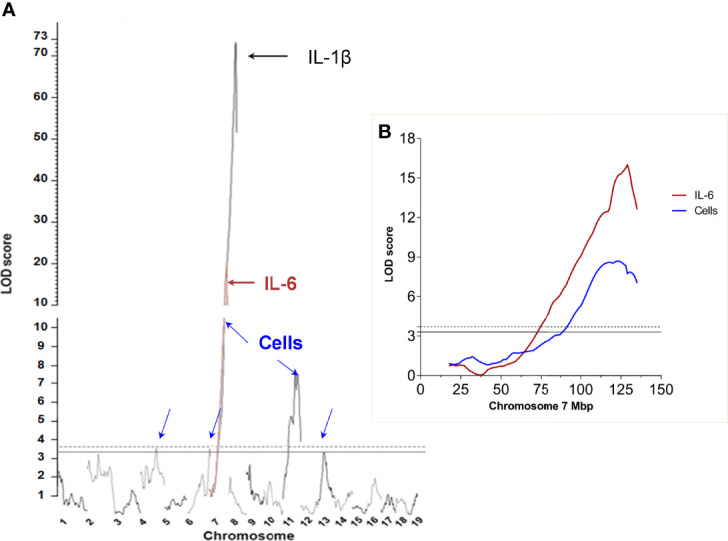
Genome wide genetic linkage analysis of loci affecting inflammatory response phenotypes in (AIRmax x AIRmin)F2 mice: **(A)** Number of infiltrating leukocytes (Cells) blue arrows, IL-6 concentration in Biogel-induced inflammatory exudate (red line and brown arrow) *ex vivo* IL-1β production by blood leukocytes stimulated with LPS and ATP (black arrow). **(B)** Detail of the peak LOD score for IL-6 levels (brown curve) and number of infiltrated cells (blue curve) at chromosome 7. Significance thresholds at α= 0.1(continuous horizontal line) and α=0.05 (dashed horizontal line).

To increase the power of the statistical association between the *Irm1* locus and IL-1β production observed in our previous work (10) we carried out an additional linkage analysis between (AIRmax x AIRmin)F2 genotype and inflammatory phenotypes, but in a larger pedigree including 693 (AIRmax x AIRmin)F2 mice. The results confirm and strengthen the mapping of the major *Irm1* locus controlling inflammation in chromosome 7, which was mapped in our earlier study (10). The LOD score peak for cell influx in inflammatory exudate increased from 3.61 to 15.74 and the LOD score for IL-1β production increased from 9.35 to 72.7 in the present F2 population. Thus, the large pedigree size allowed for a higher resolution mapping that narrowed the candidate region from 14 Mb to ~4 Mb in length, between rs13479505 (124486166 bp) and rs32381191 (128414876 bp), which includes 125 genes. IL-6 concentration in inflammatory exudates, which had not been tested in the previous analysis, showed a single and highly significant association (LOD score=18.4) overlapping the *Irm1* locus region (**Figures 1A, B**). Besides the *Irm1* locus, there are significant linkage signals for infiltrating cell numbers in chromosomes 4, 6 and 11 and one that reached suggestive level in chromosome 13.

### BC07158, Pycard, Itgam and Rgs10 Genes Are the Putative Candidate Genes of Irm1 Locus

The genomes of AIRmax and AIRmin mice have not been sequenced. Therefore, detailed SNP information is not available for these strains. In order to identify DNA variants inside the *Irm1* locus, the narrowed *Irm1* locus interval (~4Mb) was sequenced in 16 (AIRmax x AIRmin)F2 mice showing extreme phenotypes, that is, 8 with the highest (>5000 pg/mL) and 8 with the lowest (<100 pg/mL) levels of IL-1β production by blood leukocytes after LPS + ATP treatment, respectively.

**Figure 2:**
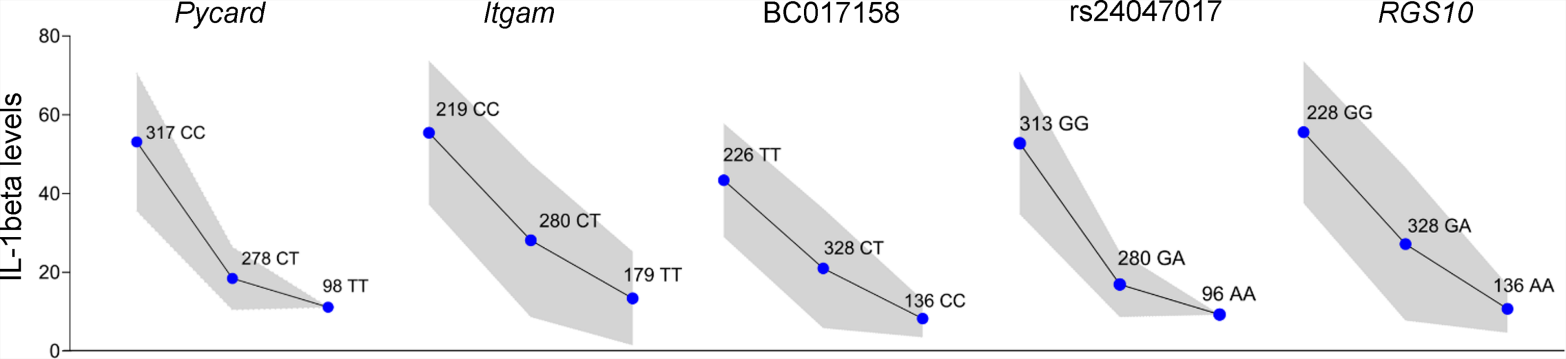
*Irm1* locus on chromosome 7 modulates IL-1 β production in (AIRmax x AIRmin)F2 mice. Allele-dosage effect of the genotypes at *Irm1* locus on the IL-1β production (square root transformed) by blood leukocytes stimulated with LPS and ATP tested in the whole (AIRmax x AIRmin)F2 population. Values expressed as means and SD. ANOVA with Tukeys posttest. –log*P* ~ 90 for all markers.

The analysis of deep sequencing genotyping data identified 14 single nucleotide polymorphisms (SNPs) with association *P* values with the Il-β levels of p < 7.49x10^-8^ and False Discovery Rate (FDR) < 1x10^-4^ between the two groups and the target region was narrowed to approximately 420 Kb. Among these SNPs, one is located in the intronic region of *BC01715*8, two in the intronic region and one in the 3’-UTR of the *Itgam* gene, one in the *Pycard* coding region, one in the intronic region of *Rgs10* and eight in intergenic regions (**Table 1**). Linkage analysis in the (AIRmax x AIRmin)F2 population, run after inclusion of these SNPs, increased the LOD score to 92 for IL-1β and did not change the LOD score for number of infiltrating cells (Cells) or IL-6 levels in Biogel induced inflammatory exudates. **Figure 2** shows the dosage effect for IL-1β of the *Irm1* locus candidate genes alleles analyzed in all 693 (AIRmax x AIRmin)F2 mice.

### Silencing of BC017158 Reduces IL-1β Production

To understand whether the genes presenting polymorphisms are involved in the modulation of IL-1β production, we carried out an *in vitro* experiment in J774A.1 mouse macrophage cell line. We silenced *BC017158, Itgam, Pycard* and *Rgs10* expression in J774A.1 cells and then we measured mRNA levels by RT-qPCR and IL-1β release by ELISA in the supernatant of silenced and of unsilenced cells stimulated with LPS and ATP. Unfortunately, *Pycard* silencing was not so effective (i.e. we observed a less than 2-fold down regulation). The results of this experiment showed that the silencing of *BC017158* gene determined a reduction of IL-1β levels (P<0.001) when compared to unsilenced cells, whereas no differences were observed in the levels of IL-1β production between unsilenced cells and cells silenced for *Itgam* and *Rgs10* (**Table 2**; **Supplemental Figure 1**).

**Table 2:** mRNA levels after gene silencing in J774A.1 cells and IL-1β production after stimulation with LPS + ATP. Results were from two independent experiments.

Gene	Relative quantity* (mean ± sd)	*P*-value ^	IL-1β# (mean ± sd)	*P*-value ^
*BC017158*	0.2 ± 0.06	0.008	0.02 ± 0.01	0.0001
*Itgam*	0.04 ± 0.07	0.008	0.8 ± 0.08	0.06
*Pycard*	0.6 ± 0.04	0.05	0.6 ± 0.02	0.001
*Rgs10*	0.05 ± 0.07	0.06	0.5 ± 0.07	0.06

*mRNA levels calculated using control cells as reference; sd, standard deviation.

Ratio between IL-1β levels measured in silenced and in control cells.

^ One-way ANOVA P-value.

### Pycard Genotypes Affect Inflammasome Driven IL-1β Production and Caspase-1 Activation in AIRmin Mice

Next we carried out a series of *in vivo* and *in vitro* experiments for the functional analysis of the *Pycard* C 127.993.599 T polymorphism. Analysis of the frequency of this SNP in the parental AIRmax and AIRmin lines, revealed that the *Pycard* (C) allele was fixed in homozygosis in AIRmax mice during the selection process whereas the frequency of the T and C alleles are 61% and 39%, respectively, in AIRmin mice. We then produced AIRmin sublines bearing the 3 *Pycard* genotypes *Pycard* ^C/C^, *Pycard* ^C/T^ *and Pycard* ^T/T^ by genotype-assisted mating. With these sublines we could evaluate the effect of *Pycard* variants on inflammatory response and IL-1β production under the influence of AIRmin genetic background.

Genotypes of the 4 genes mapping at the *Irm1* locus in AIRmax and AIRmin mice and in the AIRmin sublines are presented in **Table 3**. The three AIRmin sublines are homozygous for the AIRmin derived predominant alleles at *BC017158*, *Itgam* and *Rgs10*, irrespective of the *Pycard* genotypes.

**Table 3:** Genotypes of AIRmax and AIRmin mice and of AIRmin sublines of the 4 genes mapping at *Irm1* locus.

Mice		Genotypes			
		*Pycard/ASC*	*Itgam*	*BC017158*	*Rgs10*
AIRmax	CC		CC	TT	GG
AIRmin*	4CC:5CT:12TT		1CC:5CT:15TT	1TT:5TA:15AA	1GG:5GA:15AA
AIRmin*^Pycard^* ^C/C^	CC		TT	AA	AA
AIRmin*^Pycard^* ^C/T^	CT		TT	AA	AA
AIRmin*^Pycard^* ^T/T^	TT		TT	AA	AA

Number of mice: AIRmax (10♂ and 13♀); AIRmin (11♂ and 10♀); AIRminCC (4♂ and 4♀) AIRmin CT (4♂ and 4♀); AIRmin TT (4♂ and 4♀).

*The frequency of the AIRmax Pycard alleles in AIRmin mice is 31% and of the AIRmax Itgam, BC017058 and Rgs10 alleles in AIRmin mice is 17%.

**Figure 3:**
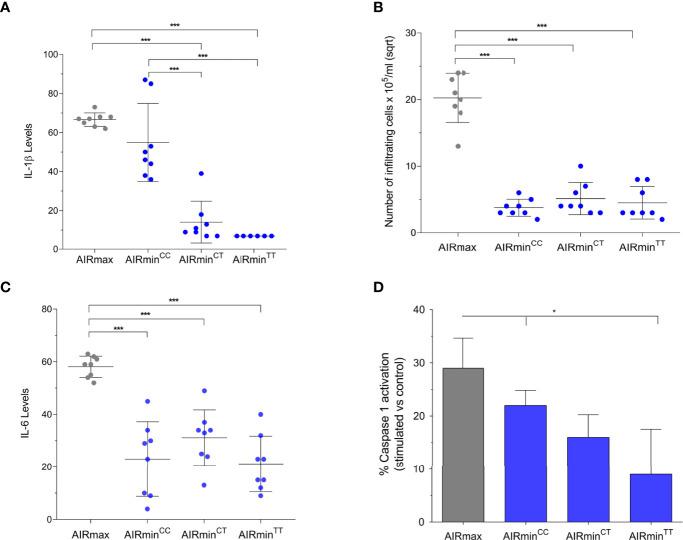
Inflammatory phenotypes in AIRmax mice and in AIRmin sublines. **(A)** IL-1β levels in plasma after stimulation of blood cells with LPS and ATP; **(B**, **C)**, number of infiltrating cells and IL-6 concentration in 24h inflammatory exudate induced by sc injection of Biogel P-100. **(D)** Caspase 1 activation in peritoneal cells stimulated with 1 μg/mL LPS for 3h and 5mM ATP for 30 min. IL-1β, IL-6 concentration (pg/mL) and cell numbers/mL exudate values were sqrt transformed, values expressed as means and SD. ANOVA test with Tukeys posttest. ***P<0.0001 and *P<0.05 difference between *Pycard* C/C and *Pycard* T/T genotypes for Caspase1.

Inflammatory response was measured in AIRmax and in the three AIRmin sublines by the number of infiltrating cells and IL-6 concentration in the Biogel-induced 24h exudate and of the *ex vivo* IL-1β production by circulating leukocytes after LPS and ATP inflammasome activation (**Figure 3**). IL-1β levels were similar in AIRmax (4.5 ± 0.4 ng/mL) and AIRmin*^Pycard^* ^C/C^ (3.4 ± 2.4 ng/mL) mice, although this last group presented increased variation in secreted IL-1β probably due to interference from other genes of the *Irm1* locus or from their genetic background. AIRmin*^Pycard^* ^C/T^ produced 0.3 ± 0.5 and AIRmin*^Pycard^* ^T/T <^0.05 ng/mL IL-1β (**Figure 3A**). It is of note that the difference between AIRmax and AIRmin*^Pycard^* ^C/C^ reached the significance level of p<0.1 suggesting that their responses are different. However, we emphasize that the most important result of this experiment is the up regulation of Il-1β production caused by the *Pycard* WT allele in homozygosis in AIRmin mice. On the other hand, leukocyte influx and IL-6 levels in inflammatory exudates were similar in the 3 AIRmin sublines and significantly different from AIRmax (**Figures 3B, C**). Accordingly to IL-1β results, the presence of the *Pycard* C allele in homozygosis resulted in higher Caspase-1 activation when compared to mice carrying the homozygous *Pycard* TT genotype (**Figure 3D**). Again AIRmin*^Pycard^* ^C/C^ mice presented a lower caspase-1 activity than AIRmax, but a higher activity when compared with the AIRmin *^Pycard^* ^T/T^ mice (p<0.05). These results point to the major effect of *Pycard* alleles in inflammasome activation and IL-1β production, without influencing the Biogel-induced acute inflammatory reaction.

### Pycard Genotypes Affect Tumor Development in AIRmin Mice

**Figure 4:**
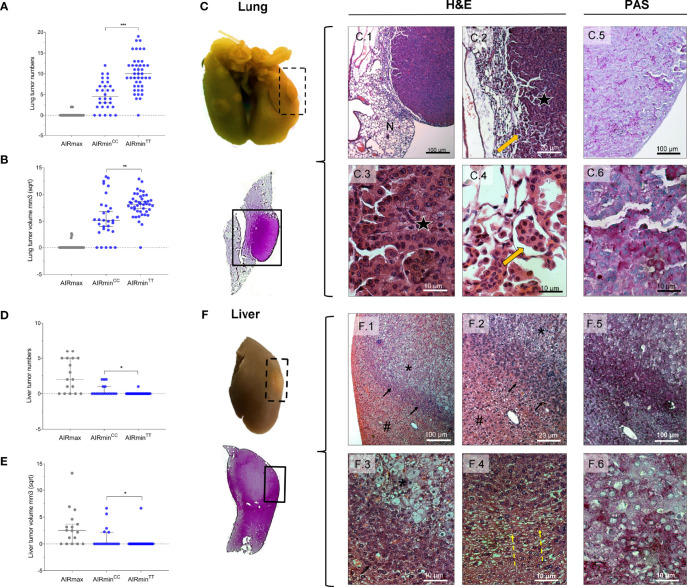
Urethane induced lung **(A–C)** and liver **(D–F)** tumors in AIRmax*^Pycard^* ^C/C^, AIRmin *^Pycard^* ^C/C^ and AIRmin *^Pycard^* ^T/T^ mice. In the vertical axis the individual tumor multiplicity **(A, D)** or the individual total volume of tumors in mm^3^ **(B, E)**. Hystopathological analysis of lung and liver tumors. **(C)** AIRmin mouse lung showing multiple nodules distributed between the right and left lobes. Tumor cellularity reveals a well-differentiated *in situ* adenocarcinoma with extensive areas with glandular arrangement (star) and invasive lesions originating from the large bronchi (C.1-3) with the presence of neoplastic emboli (arrow) in the blood vessel lumen (C.4). PAS staining shows excessive mucin accumulation in approximately 50% of the neoplastic area, characterizing mucinous adenocarcinoma (C.5-6). (F) AIRmax mouse liver showing a nodule on a lobe surface: microscopic analysis reveals a well-delimited neoplastic mass with histomorphology characteristic of hepatocellular carcinoma. Cellularity and progression of the neoplastic process (F.1-4). Transition and infiltration of neoplastic hepatocytes, at higher magnification the presence of steatosis (*), nuclear atypia and anaplasia (arrow) of neoplastic hepatocytes (F.3) and cells in the form of rowed cords (dotted arrows), mimicking normal hepatocytes (#) confirming the diagnosis of hepatocarcinoma (F.4). PAS staining shows glycogen accumulation in purple-magenta stained neoplastic hepatocytes suggesting excessive production (F.5-6). H&E and PAS; Bar 10-20-100 μm. Groups of 17♂ and 21♀ AIRmax. 17♂ and 13♀ AIRmin ^C/C^, and 26♂ and 17♀ AIRmin^T/T^ mice were injected with urethane (300 mg/Kg bw) 7 days after birth and tumors were scored at 270 days. Liver tumors develop in male mice only. Tumor volume values were sqrt transformed. Median and 95% Confidence Interval are indicated in graphs. *Mann-Whitney U test* comparing AIRmin^C/C^ and AIRmin^T/T^ mice. *p<0.05, **p<0.01, *** p<0.001.

AIRmax mice are resistant and AIRmin are susceptible to the development of lung tumors induced by the carcinogen urethane (18). On the other hand, AIRmax are susceptible and AIRmin are resistant to developing liver tumors (19). We then analyzed the possible role of *Pycard* genotypes in the behavior of the AIRmin sublines carrying WT or mutated gene alleles. The animals were treated at 7 days after birth with urethane (300 mg/Kg bw) and tumors in internal organs were analyzed at 270 days. In this experiment we observed a significant reduction in the multiplicity and total volume of lung tumors in AIRmin*^Pycard^* ^C/C^ when compared to AIRmin*^Pycard^* ^T/T^ mice (**Figures 4A, B**). Accordingly, AIRmin*^Pycard^* ^T/T^ were more resistant to developing liver tumors than the AIRmin*^Pycard^* ^C/C^ mice (**Figures 4D, E**).

In the macroscopic examination of the lung tissue, it is possible to observe five nodules distributed between the right and left lobes; in the microscopic examination the tumor distribution is 88% of the lobular area (**Figure 4C**1). The histopathological examination reveals an adenocarcinoma *in situ* well differentiated with extensive areas with glandular arrangement and invasive lesions originating from the large bronchi (**Figure 4C2-3)**. The presence of neoplastic emboli in the lumen of blood and lymph vessels suggests possible metastasis (**Figure 4C4)**. PAS staining shows excessive mucin accumulation in approximately 50% of the neoplastic area, characterizing mucinous adenocarcinoma (**Figure 4C5-6)**.

Macroscopic histopathological examination of the liver tissue shows a nodule in the hepatic lobe (**Figure 4F**) and microscopic analysis reveals a well-delimited neoplastic mass with histomorphology characteristics of hepatocellular carcinoma (**Figure 4F1)**. In detail, it is possible to see the transition and infiltration of neoplastic hepatocytes (**Figure 4F2)** and at higher magnification, the presence of steatosis, nuclear atypia and anaplasia of neoplastic hepatocytes (**Figure 4F3)**. The composition of these cells in the form of cords, lined up mimicking normal hepatocytes, confirms the diagnosis of hepatocarcinoma (**Figure 4F4)**. PAS staining shows glycogen accumulation in purple-magenta-stained neoplastic hepatocytes suggesting excessive production (**Figure 4F5-6)**.

We thus observed a modulation of carcinogenesis due to *Pycard* genotypes both at the incidence and at the multiplicity and size of malignant tumors.

### E19K Mutation at the Pyrin Domain of Pycard Interferes in ASC Specks Formation

**Figure 5:**
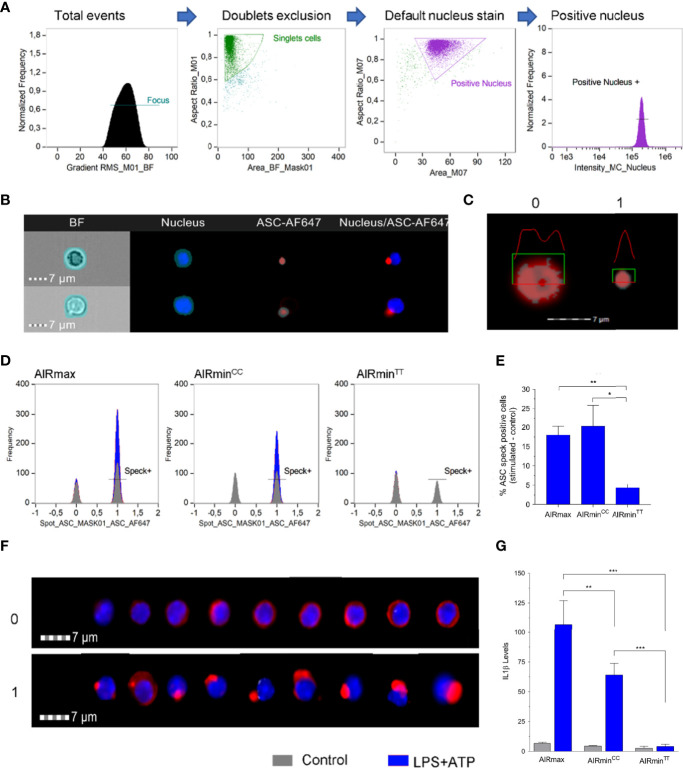
ASC-speck formation in LPS + ATP stimulated PBMCs from AIRmax*^Pycard^* ^C/C^, AIRmin*^Pycard^* ^C/C^ and AIRmin*^Pycard^* ^T/T^ mice. **(A)** Gate strategy for selected nucleus positive events in the Amnis ImageStream Imaging Flow Cytometer equipment from Luminex. **(B)** Mask definitions for the channels: BF – Brightfield, Nucleus stained with Hoechst, ASC protein stained with AF647. **(C)** Detail of the mask Spot_ASC_mask01 applied in the ASC-AF647 channel showing differences between 0 – ASC diffuse into cytoplasm and 1- ASC speck. **(D)** Representative histograms showing the positive (1) or negative (0) ASC speck cells in control (gray) or LPS+ATP stimulated (blue) PBMC from AIRmax, AIRmin*^Pycard^* ^C/C^ and AIRmin*^Pycard^* ^T/T^ mice. **(E)** Percentage of ASC specks containing stimulated cells (LPS+ATP) minus the unstimulated cells **(F)** Images of total events obtained from different regions of histogram to represent the characteristics 0 (ASC diffuse) or 1 (ASC speck). **(G)** IL-1β levels in supernatants of PBMCs from AIRmax, AIRmin*^Pycard^* ^CC^ and AIRmin*^Pycard^* ^TT^ activated with LPS+ATP or controls. IL-1β values in pg/mL were sqrt transformed. ANOVA with Tukeys posttest, *p<0.05, **p<0.01, ***p<0.001.

The results indicate that the divergent IL-1β production through NLRP3 inflammasome activation between AIRmin and AIRmax mice can be traced to a single point mutation C 127.993.599 T at the *Pycard* gene. This mutation leads to the substitution of glutamic acid to lysine at residue 19 (E19K) in the Pyrin domain of the protein which is important for the assembly of the ASC speck (16). To assess the influence of the E19K mutation in ASC speck formation, Ficoll-Paque isolated blood leukocytes from AIRmax, and of AIRmin*^Pycard^* ^C/C^ and AIRmin*^Pycard^* ^T/T^ sublines were stimulated with LPS and ATP. Leukocytes were stained for ASC and analyzed by imaging flow cytometry (**Figure 5**). DAPI stained nucleated cells were separated by the presence or absence of ASC specks (**Figures 5D-F**). Controls are cells not stimulated with LPS and ATP. The speck positive cells (1) that appear in these control cells are represented in gray color. The ASC speck positive cells that appear as a result of the stimulus are shown in blue. Inflammasome stimulation induced the appearance of large numbers of ASC speck positive cells in AIRmax and in AIRmin*^Pycard^* ^C/C^ (blue curve **Figure 5D**), with high levels of secreted IL-β (**Figure 5G**) whereas in AIRmin*^Pycard^* ^T/T^ preparations, ASC speck positive cell number is similar to that of controls (grey curve). The difference in IL-1β production between AIRmax and AIRmin*^Pycard^* ^C/C^ is significant (p<0.01), confirming the results presented in **Figure 3A**. IL-1β levels in homozygous animals for the mutation did not reach the detection limit of the assay (10pg/mL) **Figure 5G**.

### Computational Site Directed Mutagenesis Analysis

**Figure 6:**
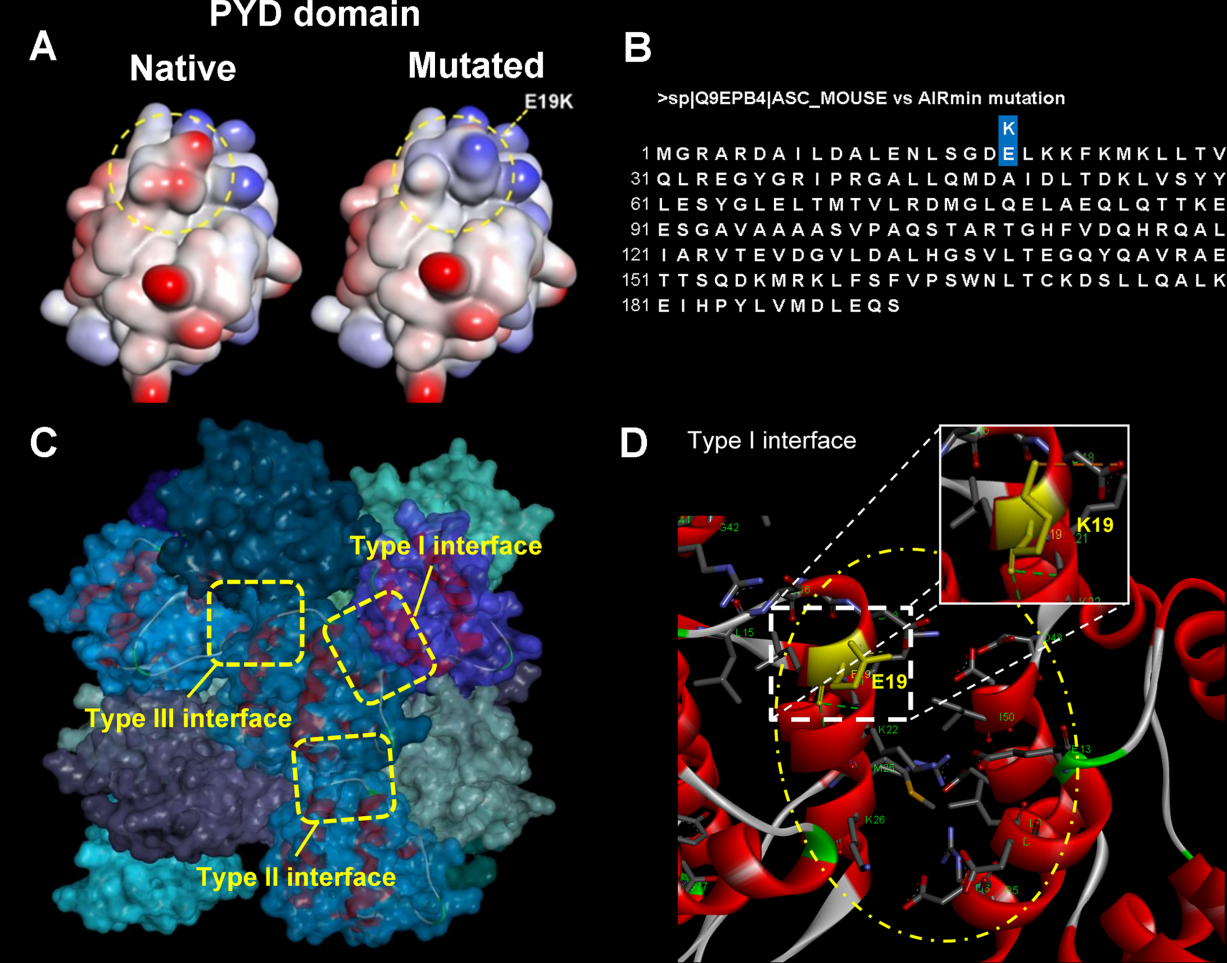
Computational modeling of site directed E19K mutation at PYD domain of ASC protein in mouse and ASC speck formation in J774A.1 cells overexpressing WT or mutated E19K ASC. **(A)** Interpolated charge surfaces of ASC PYD in native and E19K mutated monomers (blue color: positively charged region; red color: negatively charged region; white color: non-charged region). **(B)** ASC protein amino acid sequence with the indication of the E19K substitution. **(C)** 3D arrangement of the ASC-PYD filament [PDB ID 2N1F, fifteen monomers (14)]. Side view of the ASC-PYD filament in molecular surface representation. Three asymmetric interaction interface types, I-III, which are characteristic for the filament architecture, are indicated by dashed square rectangles. The E19 residue is located at the type I interface region. **(D)** Electrostatic interactions established in the type I interface (yellow oval form; subunits/monomers H and G). The amino acid residues are shown in stick models, where carbon atoms are in gray color, oxygens in red, nitrogen atoms in blue, sulfur in orange, and hydrogens in white; the protein subunits are in ribbon representation (Discovery Studio Visualizer 4.0; Accelrys Software Inc., 2005-13). We present two different fields (upper and lower) for each cell preparation that were observed at 5 and 120 minutes after the stimulus.

The PYD N-terminal domain of each monomer from the mouse ASC protein presents 6 alpha helices and forms a helical filament in which each PYD interacts with six others through type I, II and III interfaces (PDB ID 2N1F (16) (**Figure 6C**). The amino acid residues from helices 1 and 4, on subunit/monomer H, and from helix 3, in monomer G, are placed in the type I interface. Regarding type I interface, electrostatic interactions between residues of opposite charges (salt bridges, for instance; **Figure 6D**) are established contributing to filament stabilization (16). The positively charged side chain of residues K21, K22, K26 (helix 2), and R41 (helix 3), on monomer/subunit H, as well as the negatively charged side chain of residues D6, D10, E13 (helix 1), D48, D51, and D54 (helix 4), on monomer/subunit G, are involved in those electrostatic interactions. It is noteworthy that the E19K mutation is placed in a region defined as type I interface. The computational model obtained from site directed mutagenesis analysis revealed that the E19K mutation found in AIRmin mice (gene sequence in **Figure 6B**) does not significantly change the conformational arrangement, but it does change the electronic density distribution regarding the type I interface (**Figure 6A**). In this regard, the E19K mutation would be predicted to impair filament formation. On the other hand, according to molecular docking simulations run by our research group (data not shown), the E19K mutation seems to not interfere in the interaction process of the PYD domain with the NLRP3 receptor.

### PYCARD E19K Substitution Impairs ASC Speck Formation in J774A.1 Cells

We used CRISPR to knock out the *Pycard* gene in J774A.1 cells (**Supplemental Figure 2**). While non-edited J774A.1 cells produced 600 pg/mL IL-1β after stimulation with LPS and ATP, IL-1β levels in supernatants of *Pycard* deficient stimulated cells were below the detection limit of the ELISA method.

Retroviral transduction was then used to generate J774A.1ASC^+/-^ cells stably expressing fluorescent WT or E19K mutated ASC, in order to interrogate the interference of the E19K polymorphism on ASC speck formation after NLRP3 inflammasome activation, without the interference of AIRmin genetic background. FACS sorted cells were stimulated with LPS and ATP and images were taken in time lapse at HCS (*High Content Screening*). ASC expression levels varied more in cells transduced with mutated form (**Supplemental Figure 4C**), so we looked for fields where the expression was similar in the two preparations. In these we observed that the percentage of cells with specks is similar at the beginning of the reaction but the specks in several cells bearing the mutated form of ASC do not persist over time.

**Figure 7:**
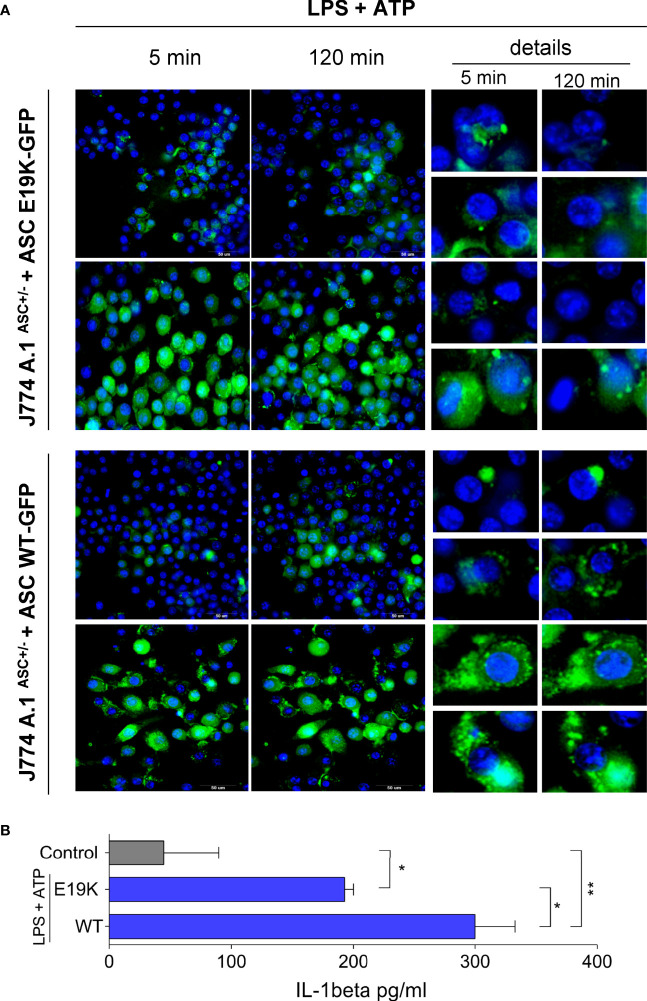
ASC speck formation in J774A.1ASC ^+/-^ cells stably expressing WT ASC or mutated E19K ASC linked to GFP after stimulation with LPS (1μg/mL for 3 h) and 5mM ATP. **(A)** ASC specks formation 5 min and 120 min after ATP addition. In detail the appearance and evolution of ASC specks, which are small and tend to disappear with time in cells expressing the mutated form, and apparently are more frequent and persistent in cells overexpressing the WT *Pycard* allele. Images were taken in two different fields (upper and lower) for each cell preparation at 5 and 120 minutes after ATP addition in High Content Screening (HCS) equipment. **(B)** IL-1β secretion (pg/mL) by cells after stimulation with LPS (1 ug/mL for 3h) and ATP (5mM ATP for 1h), mean and SD, ANOVA with Tukeys posttest *P<0.05, ** P<0.01.

Otherwise, specks were seen in WT cells that persisted up to 120 min after stimulation with ATP (**Figure 7**). We also observed an increase in ASC expression at times after the stimulus.

The two preparations of cells transduced with both forms of the gene produced IL-1β, but the levels were significantly higher in the cells transduced with the wild type f *Pycard* allele (**Figure 7B**).

## Discussion

The aim of the present study was to reduce the *Irm1* locus interval at chromosome 7 mapped in our previous study (10) to identify candidate genes regulating the acute inflammatory response and IL-1β production. The large size (693 mice) of the (AIRmax x AIRmin)F2 pedigree used here, allowed a high resolution mapping that reduced the *Irm1* candidate region to 4 Mb in length, including 125 genes. This locus regulates inflammasome-induced IL-1β production by circulating leukocytes, as well as the local leukocyte influx and the level of IL-6 in the inflammatory exudates induced by Biogel. The mapping of the IL-6 regulation to the *Irm1* locus, confirms its importance in the modulation of acute inflammation. In addition, other significant linkage signals for leukocyte influx were mapped in chromosomes 4, 6, 11 and a suggestive signal mapped in chromosome 13, demonstrating the multigenic control of this complex phenotype.

Deep sequencing of (AIRmax x AIRmin)F2 mice groups presenting extreme and opposite IL-1β levels narrowed the *Irm1* locus region controlling this phenotype to 420 Kbp containing four genes. Through *in vitro* investigation of these genes, we individuated *BC017158* as a possible *Irm1* candidate able to reduce IL-1β levels when silenced in a macrophage cell line and *Pycard* the only gene that presented a missense mutation (exon 1) which impaired IL-1β production and ASC specks formation in AIRmin mice.

Regarding the role of *BC017158* our results demonstrated that it’s silencing in J774.1 cells strongly reduced IL-1β levels. Moreover, the identification of a SNP in an intronic region of *BC017158*, led us to hypothesize that this SNP could act by modulating the level of protein expression or the formation of an alternative protein isoform. Little information is available in literature about *BC017158* functions; this gene is orthologous to human RUS family member 1 (*Rusf1*) that codes for a putative transmembrane protein that may be involved in protein-protein interaction. There are several microRNAs that interact with different BC017158 transcripts, possibly modulating their expression levels. One example is muMir155 which is involved in a miRNA-based regulatory network that is implicated in macrophage activation for modulating inflammatory responses (20). Our results nevertheless suggest a role of the *BC017158* gene in inflammation through regulation of IL-1β production.

Although *Pycard* candidacy for *Irm1* locus was excluded in our previous work, because the SNPs detected along the gene did not show significant frequency deviation between the AIRmax and AIRmin groups tested at that time (10), results of the present work led us to reconsider its involvement in determining the divergent acute inflammatory response in AIR lines. In the previous analysis we assumed that alleles at any loci influencing the phenotypes in AIRmax and AIRmin populations were fixed in homozygosis. However, the high degree of genetic heterogeneity of the lines does not guarantee that the genotyped SNPs close to or inside the QTL are homozygous. In the present work we carried out the analysis based on heterozygosity at the QTL within the two populations, even if the theoretical assumption of homozygosity fixation at all QTLs is correct. Here, the candidacy of *Pycard* for exerting the effects of the *Irm1* locus is supported by the deep sequencing of (AIRmax X AIRmin)F2 mice showing extreme high or low IL-1β levels after LPS + ATP treatment. We identified a SNP in the first exon of the *Pycard*, thus suggesting that the two *Pycard* alleles could differently modulate IL-1β levels in AIRmax and AIRmin mice. This was confirmed *in vivo* by the up regulation of IL-1β production that we observed in stimulated blood leukocytes from AIRmin*^PycardC/C^* mice, homozygous for the AIRmax *Pycard* WT C allele, compared to AIRmin*^PycardT/T^*. This effect can be attributed to the *Pycard* variants since the 3 AIRmin sublines: AIRmin*^PycardC/C^* AIRmin*^PycardC/T^* and AIRmin*^PycardT/T^* are homozygous for the predominant AIRmin alleles at the other genes mapping at *Irm1* locus: *BC017158*, *Itgam* and *Rgs10*.

It is noteworthy that this *Pycard* variant does not exist among the 8 inbred lines (A/J, BALB/cJ, CBA/J, C57BL/6J, DBA2/J, P/J, SJL/J and SWR/J) used for the production of the initial population (F0) that was bidirectionally selected for the production of the AIRmax and AIRmin strains (9). It is possible that this mutation was present in some animal(s) used for mating in the foundation population (F0) or that the mutation occurred during the selection process, maybe by cytosine deamination, a very common phenomenon that results in mutations and which accounts for almost half of the known pathogenic SNPs (21).

Other authors recently described *Pycard* as the candidate gene of the locus called *Irm3*. This study, similar to our QTL mapping for IL-1β production dependent on inflammasome activation, was done in bone marrow-derived macrophages originated from crosses between AKR and DBA2 mice that differ in the quantitative production of this cytokine. *Pycard* was considered the causal candidate of the locus due to a SNP between the AKR and DBA/2 lines in the 3’ UTR region (22). AKR was not part of the 8 inbred strains that formed the initial population of the selection process of the AIRmax and AIRmin strains, however the results corroborate the importance of variants in this gene or in nearby non-coding regions for the activation of the inflammasome for IL-1β production.

PYCARD or ASC for Apoptosis-Associated Speck-like protein (ASC) containing a Pyrin and a caspase-recruitment domain (CARD) is the inflammasome complex adaptor protein. The N-terminal pyrin domain (PYD) of ASC links to NOD like receptors such as NLRP3 by homotypic PYD interactions and links to caspase-1 by homotypic C-terminal caspase recruitment and activation domain (CARD) interaction, which cleaves pro-IL1β and pro-IL18 to active forms that are then released. Caspase-1 also activates the pore-forming gasdermin D inducing cell death by pyroptosis.

ASC speck formation constitutes a suitable readout for inflammasome activation. Studies combining NMR and cryo-electron microscopy defined the structure and complex formation of PYCARD specks (16). ASC undergoes PYD-dependent oligomerization with successive ASC proteins forming filaments that condense into macromolecular structures. ASC PYD is polar and self-interaction is specifically based on and stabilized by amino acid residues having opposite charges in each side of two subunits (type I interface). E13A, E19A, K21A, K26A, R41A, D48A, D51A, L68A, L73A, K21A and K26A mutations were induced in PYCARD PYD domain and have shown the importance of those residues for the PYD-PYD interaction and, consequently, for the ASC speck formation (23–25). Despite the fact that several residues participate in the interaction interface between ASC PYD subunits and, therefore, some changes can be tolerated, studies have demonstrated that a proper balance of the charged residues in the interaction type I interface is important for filament formation. Herein, we have provided data to suggest that the naturally occurring ASC PYD domain mutation, E19K, detected in AIRmin mice, resulting in the substitution by a positively charged Lysine residue is likely disruptive to the correct formation of ASC specks.

In fact, we could observe this effect by the higher number of ASC positive cells in stimulated blood leukocytes from AIRmax or AIRmin*^PycardC/C^* compared with AIRmin*^PycardT/T^* mice. The results show that the expression of the AIRmax derived *Pycard* allele in the AIRmin genetic background clearly interferes with inflammasome activation and ASC speck formation. Furthermore, we got a similar picture when the two variants of the gene were overexpressed in J774A.1 cells. After inflammasome stimulation with LPS + ATP, J774A.1 cells overexpressing the *Pycard* WT allele produced ASC specks, some large ones that persist up to 120 minutes, whereas small specks that decrease over time were observed in the cells bearing the mutant allele (**Figure 7**). These are preliminary results and further in-depth studies of the actual interference of the mutation in the formation of specks are required. Our hypothesis was based in studies of other groups that clearly demonstrated the importance of residues in interface 1 of the PYD protein domain for filament formation (17–19). A deficient formation of ASC specks in AIRmin could explain a lower activation of Caspase-1, and a decreased release of IL-1β by stimulated cells. However, we observed that cells transduced with the mutated form were able to produce IL-1β indicating that the E19K is not defective, but just less functional than the wild type. Besides that, comparing the IL-1β production by J774 cells expressing the mutated ASC with the very low responses we obtained with cells from AIRmin *^Pycard^* ^C/T^ AIRmin *^Pycard^* ^T/T^ animals, we can conclude that the genetic background, or other genes located in the *Irm 1* locus, might contribute to the low response of these mice.

Inflammasome activation plays central roles in autoimmune and neurodegenerative diseases, infections and cancer (26). Besides their central role in inflammasomes for cytokine release, ASC specks can be released to the extracellular space, accumulate in inflamed tissues and can be internalized by surrounding inflammatory cells inducing inflammation chronicity or amplification, therefore worsening the disease or contributing to cure (26, 27). The AIRmax and AIRmin mouse lines differ in natural resistance to infections, to the induction of autoimmune diseases and chemical carcinogenesis as well as in tissue repair capacity (18, 19, 28–34). These phenotypes might be influenced by the differential *Irm1* locus-dependent inflammasome activation between the two lines described here. In fact, AIRmin mice bearing the AIRmax *Pycard* allele (AIRmin*^Pycard^* ^C/C^) were more resistant to the development of lung adenocarcinomas and more susceptible to developing hepatocarcinomas than AIRmin*^Pycard^* ^T/T^, following the behavior of AIRmax mice (11, 19).

In the case of the lung, it is worth noting that AIRmin mice and the two AIRmin *^Pycard C/C^* and AIRmin *^Pycard T/T^* sublines carry the lung tumor susceptibility allele at the major Pulmonary Adenoma Susceptibility 1 (*Pas1*) locus, unlike AIRmax, which have the resistance allele (11, 29). It was thus interesting to observe *Pycard* as modifier of a major cancer susceptibility gene. In the case of the liver, in previous studies with AIRmax and AIRmin mice, we mapped two loci controlling susceptibility to liver tumors (chromosomes 2 and 9) (12) and, even in the background of AIRmin, resistance was modulated by the *Pycard* CC genotype. These results suggest that polymorphisms of this gene interfere with carcinogenesis in both organs.

The main point of this study is that the AIRmax and AIRmin mice and the AIRmin*^Pycard^* ^C/C^ and AIRmin*^PycardT/T^* sublines are the only mice described in the literature where the *in vivo* phenotypic effect of a naturally occurring missense mutation of the *Pycard* gene can be evaluated and are therefore a novel research tool. Furthermore, whole genome linkage analysis using SNPs in a heterogeneous (AIRmax x AIRmin)F2 population involved *Pycard* as well as *BC017158* (*Rusf1*) in the control of IL-1β production. These genes map to the unique and extremely significant *Irm1* locus controlling NLRP3 inflammasome activation (35, 36). Further studies are needed to clarify how *Pycard* and *BC017158* genes interact in the modulation of IL-1β production.

Genes at the major *Irm1* locus as well as genes mapping to the interval of other significant QTLs evidenced in WGA analysis are candidates to contribute to the control of acute inflammatory response. Altogether, our results help in understanding the genetic mechanism of the acute inflammation process.

## Data Availability Statement

The variant data for this study are deposited in the European Variation Archive (EVA) at EMBL-EBI under accession number Project: PRJEB53276; Analyses: ERZ10780065.

## Ethics Statement

All procedures were approved by the Institutional Animal Care and Use Committee of Butantan Institute (protocol n° 3587190320), and all animals received humane care, according to the criteria outlined in the “Guide for the Care and Use of Laboratory Animals” prepared by the National Academy of Sciences and published by the National Institutes of Health.

## Author Contributions

TD and OI contributed to conception, design and writing of the manuscript. AB prepared DNA samples and contributed to linkage analysis, performed CRISPr experiments and wrote sections of the manuscript. FC contributed to linkage analysis, performed gene silencing experiments and wrote sections of the manuscript. JS performed analysis of ASC specks formation in HCS and Image stream and wrote sections of the manuscript. RP performed and organized sequencing data. BS contributed to the CRISPr experiments. MF and JJ contributed to linkage and sequencing data analysis. JJ performed the design of primers for qPCR analysis. WC and OR produced the mouse lines, performed inflammation assays and carcinogenesis experiments. MI performed caspase 1 assays and contributed to CRISPr experiments design. NS and LM contributed to inflammation, IL-1 and IL-6 analysis. GM contributed to plasmid production and purification for CRISPr experiments. CDP performed HCS analysis and contributed to CRISPr RNA guides design. SE performed hystopathlogical analysis of tumors. MG performed design of WT and mutated Pycard gene sequences for viral transduction of J774A.1 cells. KP performed molecular modeling and computational site directed mutagenesis analysis and wrote a section of the manuscript.All authors contributed to manuscript revision, read, and approved the submitted version.

## Funding

This study received funding from São Paulo Research Foundation (FAPESP) process number 2016/07007- 9 and FAPESP/GlaxoSmithKline Grant 2020/13139-0. JGS was supported by FAPESP post doc fellowship, process number 2017/06736-0; OGR, NS and JRJ were partially supported by Fundação Butantan; OCMI and MDF were partially supported by Brazilian Council for Research (CNPq), and Fundação Butantan. The funders had no role in study design, data collection and analysis, decision to publish, or preparation of the manuscript.

## Conflict of Interest

Author KP is startup founder CEO, Director of the Computer-Aided Drug Design Division of ALCHEMY – Inovation, Research & Development Ltd, University of São Paulo, Brazil.

The remaining authors declare that the research was conducted in the absence of any commercial or financial relationships that could be construed as a potential conflict of interest.

## Publisher’s Note

All claims expressed in this article are solely those of the authors and do not necessarily represent those of their affiliated organizations, or those of the publisher, the editors and the reviewers. Any product that may be evaluated in this article, or claim that may be made by its manufacturer, is not guaranteed or endorsed by the publisher.
