# Supplementary material for: Pycard and BC017158 Candidate Genes of Irm1 Locus Modulate Inflammasome Activation for IL-1β Production
Source: Front Immunol. 2022 Jun 21;13:899569. doi: 10.3389/fimmu.2022.899569 (PMC9254735; doi:10.3389/fimmu.2022.899569)
Supplement: Supplementary file 1 [file DataSheet_1.docx]

**Supplemental Table 1**

Supplemental Table 2 – TaqMan assays used to genotype SNPs in the Irm1 locus candidate genes

|  |  |  |  |
| --- | --- | --- | --- |
| Assay Name | SNP | Forward Primer | Reverse Primer |
| BC017158 | rs23194730 | GTTAGAGGGCTTCCCAGCAA | GGTGGTTTTTGAAGCCCTAAGGATT |
|  |  | Reporter 1 | Reporter 2 |
|  |  | AAATGTCGGAGCTTTT | AAATGTCGGTGCTTTT |
|  |  |  |  |
| Pycard | Novel SNP | Forward Primer | Reverse Primer |
|  | (pos. 127993599) | ACTGTCAGCAGCTTCATCTTGA | CTGGACGCTCTTGAAAACTTGTC |
|  |  | Reporter 1 | Reporter 2 |
|  |  | CTTTTTGAGTTCATCCCC | CTTTTTGAGTTTATCCCC |
|  |  |  |  |
| Itgam12 | rs50943650 | Forward Primer | Reverse Primer |
|  |  | TCAAGGCTAACCTGTGCTAAAGG | CTGCTCTATTCCACAAAGGGTTAAAGA |
|  |  | Reporter 1 | Reporter 2 |
|  |  | AGGCTTTAACCCCCCC | AGGCTTTAATCCCCCC |
|  |  |  |  |
| Rgs10 | rs32381191 | Forward Primer | Reverse Primer |
|  |  | GCCTGGGCATCTAAGAAAATGAAAG | GTGCGATGTGGCTTCAATGG |
|  |  | Reporter 1 | Reporter 2 |
|  |  | TGTCACTTGTCTATATTTT | CACTTGTCCATATTTT |
|  |  |  |  |


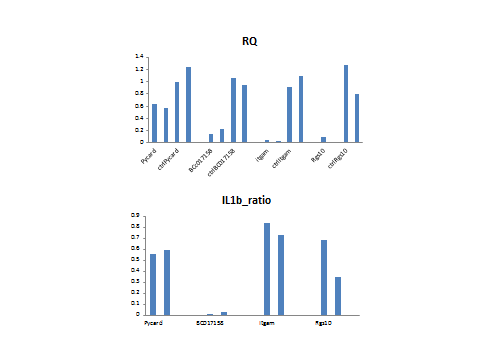


**Suppl. Figure 1**. mRNA levels before (ctrl) and after gene silencing in J774A.1 cells and IL-1β ratio between normal and gene silenced cells after stimulation with LPS + ATP. Results were from two independent experiments.


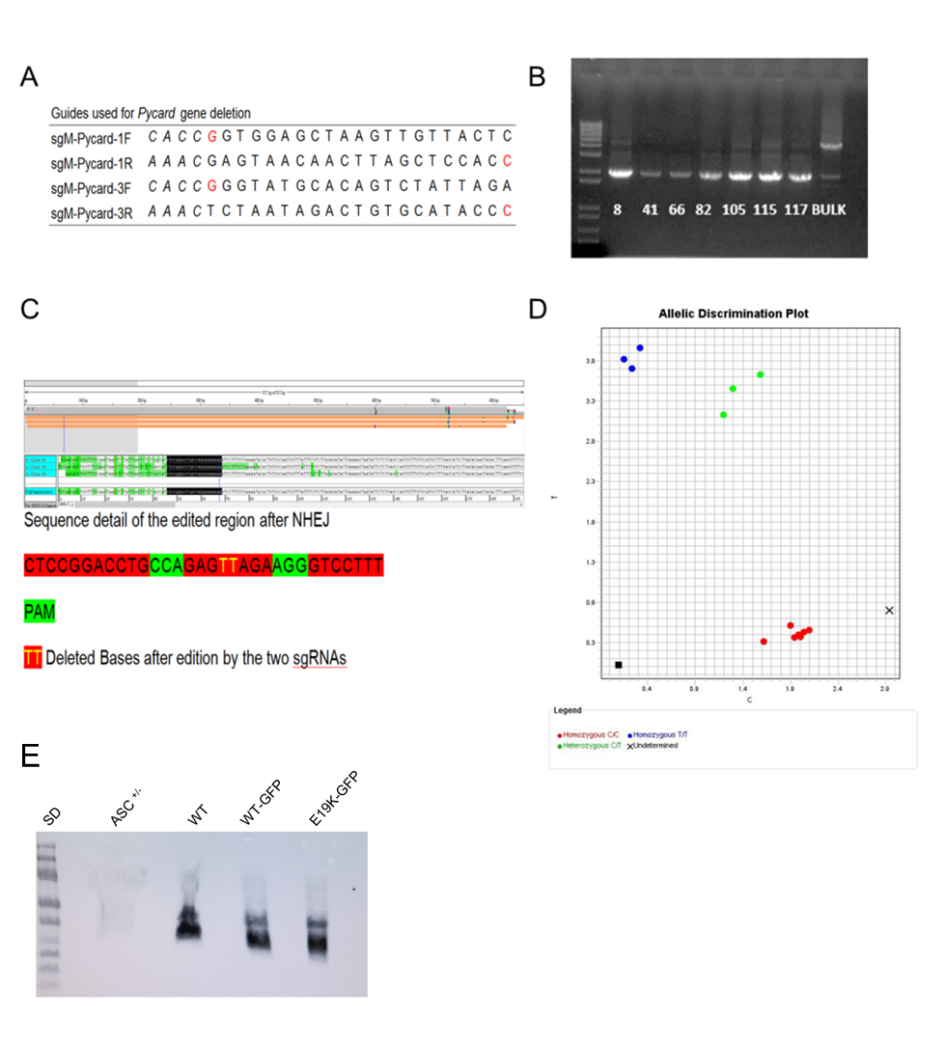


**Suppl. Figure 2.** CRISPR Cas9 editing of *Pycard* in J774A.1 cells.

A) Sequences of guides used for *Pycard* gene deletion. Position of the guides:

Guide GFP-1 - 127.593.100 - 127.593.121

Guide PURO3 - 127.590.558 - 127.590.577

GRCm39 (annotation release 109), Ensembl Release 105

B) Agarose gel electrophoresis of gDNA from 7 clones expanded after single-cell sorting of CRISPR/Cas9 edited cells (Bulk) for Pycard gene deletion. Bulk presents one band of 4460 nt showing the whole gene in non-edited cells and one ~2500 nt band for cells where gene deletion occurred. Numbers refer to Pycard edited clones (small band ~2500 nt). PCR conditions favored the appearance in the gel of the band showing the deletion. C) Sequence alignment obtained with primers flanking the Pycard edit sites in three edited clones. Highlighted in black is the region where non-homologous end joining (NHEJ) occurred. Note that two T nucleotides were deleted in the edited sites of the 3 clones (yellow font). D) TaqMan genotyping of the clones sequenced in (C) (red dots). Green and blue dots represent control DNA (T/T and C/T genotypes). Allele specific RT- PCR assays, showed that the cells are heterozygous (*Pycard*^+/-^). E) Western blot of ASC protein (β-actine normalized) in ASC^+/-^ deficient J774A.1, native J774A.1, and J774A.1 cells transduced with ASC WT GFP or ASC E19K GFP.


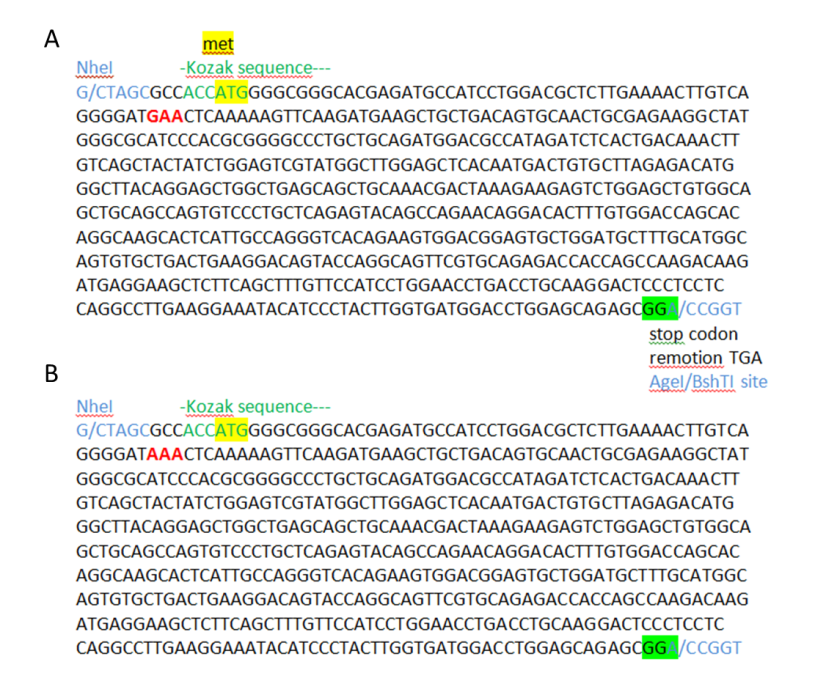


**Suppl. Figure 3** A) WT and B) mutated *Pycard* sequences for cloning in pLJM1-GFP plasmid (VETOR pLJM1-EGFP: <https://www.addgene.org/19319/>). Mutation is highlighted in bold red


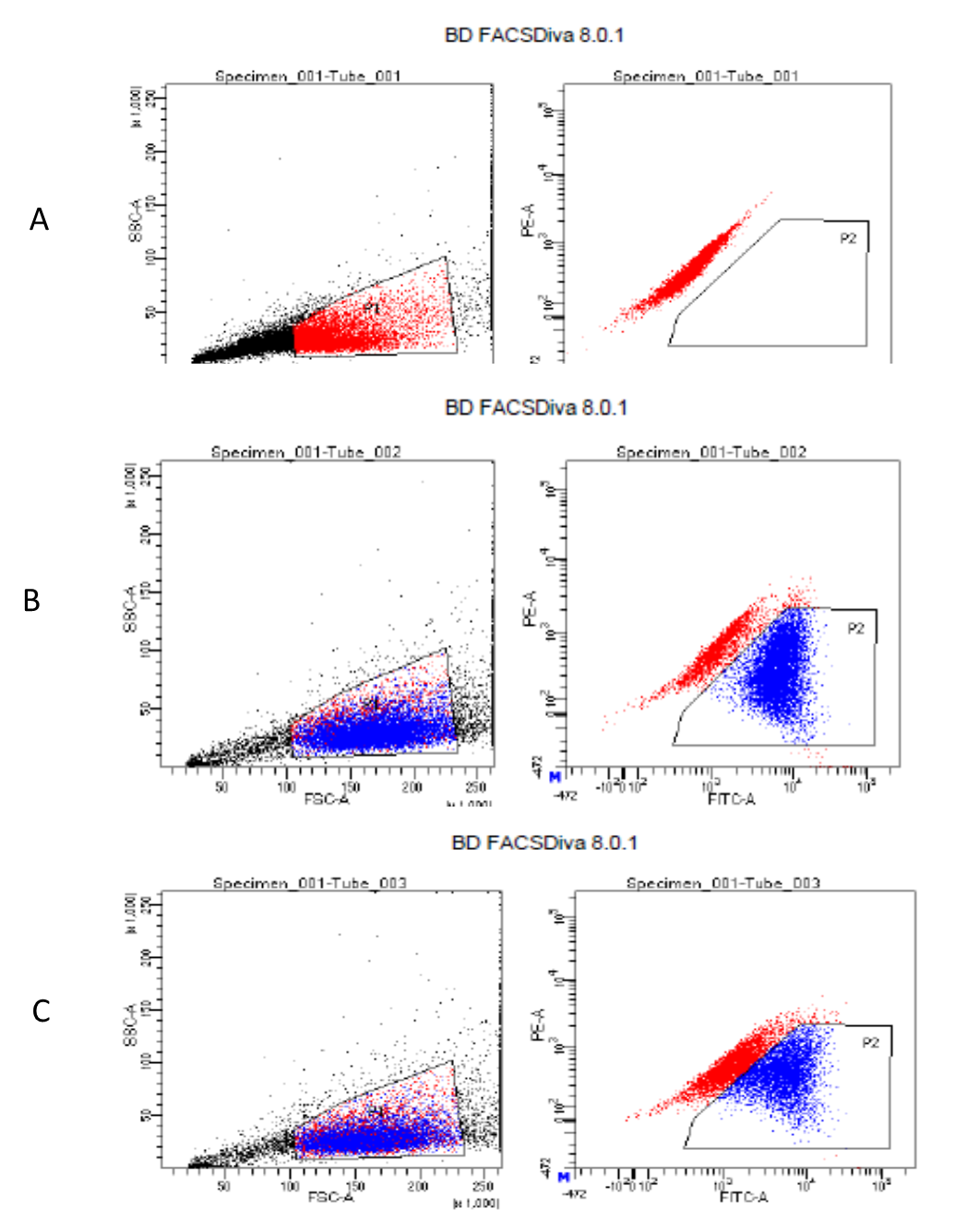


**Suppl. Figure 4.** Fluorescent sorting of cells stably expressing *Pycard* gene**.** A) Non transfected cells, B) cells transfected with WT ASC-GFP , C) Cells transfected with E19K ASC -GFP.


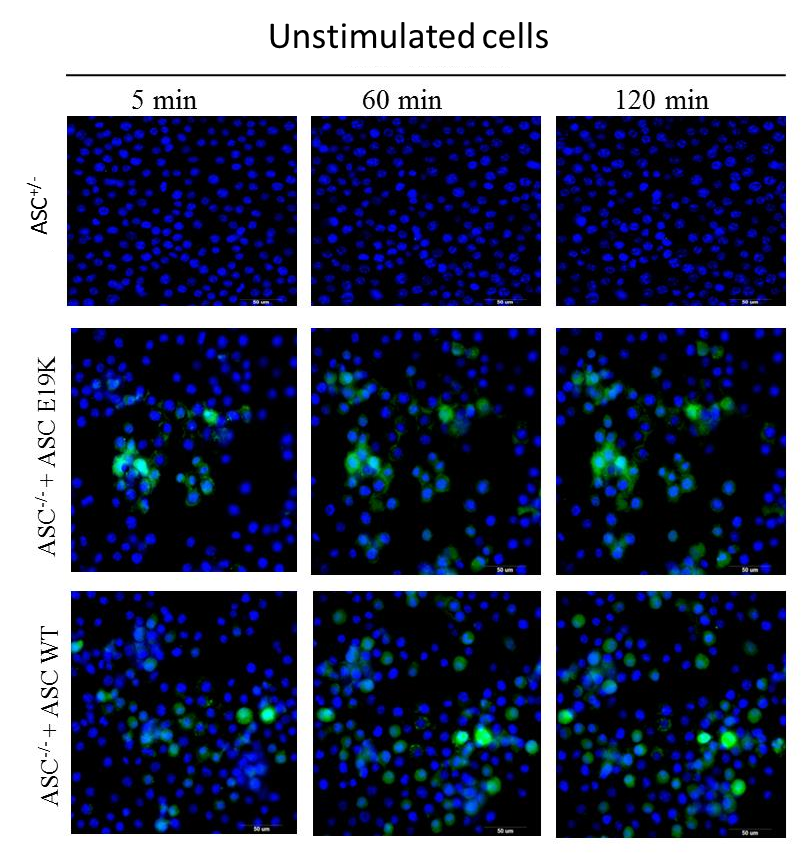


**Suppl. Figure 5.** ASC expression by unstimulated J774 A.1 ASC deficient cells. Upper line, non transduced cells; Middle line, transduction with mutated E19K ASC-GFP; Last line, transduction with WT ASC-GFP.
